# Anomalous right coronary artery managed with bypass and proximal ligation

**DOI:** 10.1186/s13019-024-02896-4

**Published:** 2024-07-13

**Authors:** Harry Ramcharran, Ahmad Nazem

**Affiliations:** 1 Department of Cardiothoracic Surgery, SUNY Upstate Medical University, 750 East Adams Street, Syracuse, NY 13210 USA; 2 Department of Cardiothoracic Surgery, St. Joseph’s Hospital, Syracuse, NY USA

**Keywords:** Anomalous origin of the coronary artery from the opposite sinus of Valsalva, Coronary artery bypass, Proximal ligation

## Abstract

**Background:**

An anomalous origin and inter-arterial course of the right coronary artery is a rare anomaly that can lead to sudden ischemic cardiac death if left untreated. We present a case of a patient with an anomalous right coronary artery originating from the left coronary sinus and an inter-arterial course that was managed with coronary artery bypass surgery using a suitable internal mammary artery conduit. The proximal right coronary artery was ligated to prevent competitive flow.

**Case Presentation:**

A 69 year-old-male with a ten-year history of intermittent chest pain and dyspnea with a negative workup underwent a cardiac catheterization, which showed an anomalous right coronary artery (RCA) originating from the left coronary sinus, with an inter-arterial course between the ascending aorta and pulmonary artery, and approximately 70% narrowing of the proximal RCA. The patient underwent an on-pump coronary artery bypass using the right internal mammary artery (RIMA) as a conduit, with segment 2 of the RCA being the target. The proximal RCA was ligated. Intra-operatively, there were no signs of ischemia or arrhythmia. The patient was successfully taken off cardiopulmonary bypass and eventually discharged home.

**Conclusion:**

Symptomatic anomalous origin of the right coronary artery with an inter-arterial course can be treated successfully with coronary artery bypass surgery with the internal mammary artery as a conduit. Ligation of the proximal right coronary artery is essential to minimize competitive flow through the bypass graft.

## Background

An anomalous origin and inter-arterial course of the right coronary artery is a rare anomaly [1]. Its inter-arterial course can vary from high inter-arterial, low inter-arterial, and hypoplastic anomalous orifice between the ascending aorta and pulmonary artery [2]. It can also be potentially fatal if left undiscovered and result in sudden ischemic cardiac death due to various proposed mechanisms ranging from mechanical compression of the anomalous artery in its inter-arterial course, acute angulation of the ostium, or vasospasm [2]. We present a rare case of an anomalous right coronary artery originating from the left coronary sinus with an inter-arterial course in an elderly patient, which was treated with a surgical bypass and ligation of the proximal right coronary artery to eliminate competitive flow [3].

## Case presentation

**Fig. 1:**
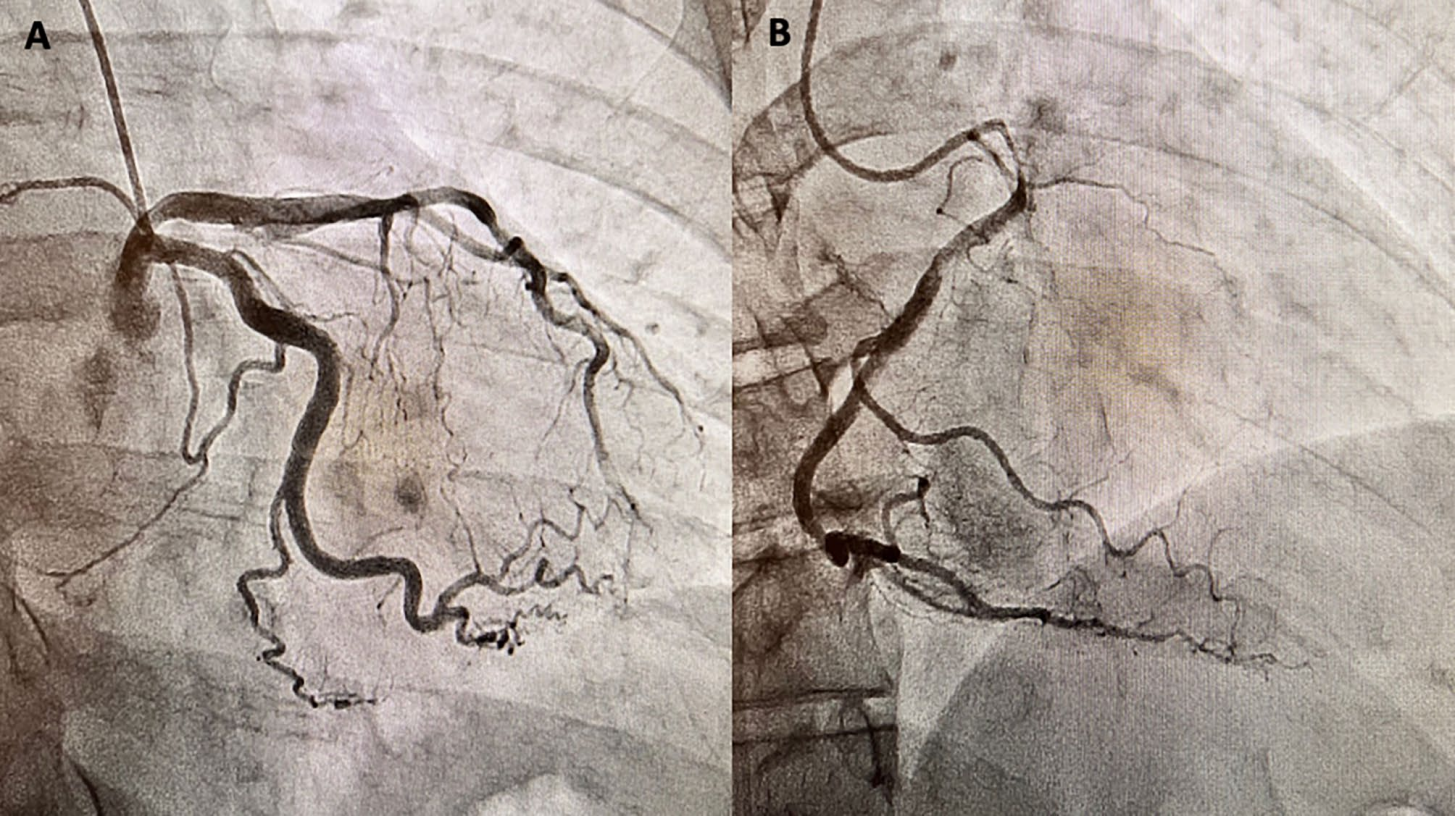
Coronary Angiogram depicting the origin of the left anterior descending artery and the left circumflex artery (**A**) as well as the origin of the right coronary artery (**B**) from the left coronary sinus

**Fig. 2:**
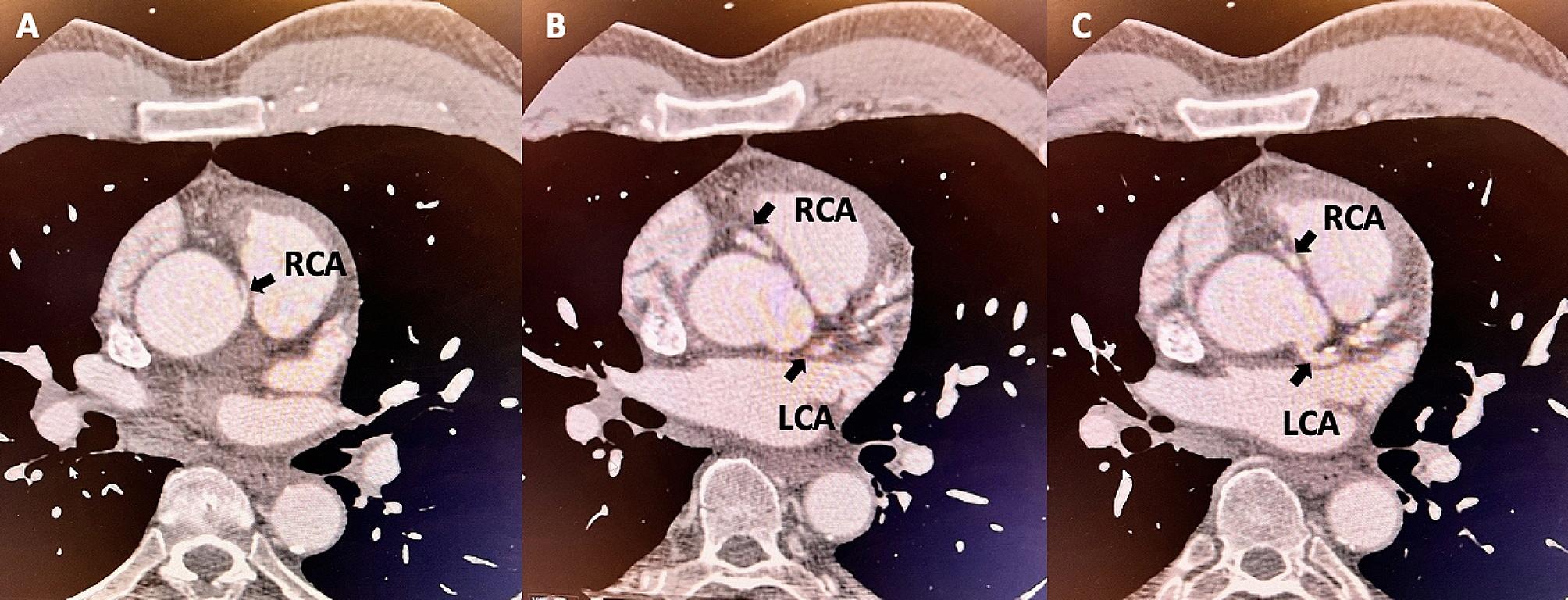
Computed Tomography Angiography of the chest showing the origin of the right coronary artery (RCA) **(A**), its inter-arterial course (**B**), and the origin of the left coronary artery (LCA) (**C**)

A 69-year-old man with a past medical history of well-controlled insulin-dependent diabetes mellitus type 2 and hypertension was referred to our clinic for exercise-induced dyspnea and chest pain. The patient had been experiencing occasional episodes of chest pain and tachycardia with mild exertion for approximately ten years. He presented to the emergency departments at multiple hospitals and had a normal workup (EKG and echocardiogram). He was referred to our clinic for further evaluation of his persistent symptoms. A thorough workup was performed and showed a normal ejection fraction without evidence of exercise-induced ischemia or arrhythmia. He ultimately underwent a cardiac catheterization, which revealed an anomalous right coronary artery (RCA) and an inter-arterial course between the ascending aorta and pulmonary artery (Fig. 1). Computed Tomography Angiography of the chest revealed the RCA originating above the left coronary sinus. Approximately 70% narrowing of the proximal RCA was also noted with an acute angulation of the origin (Fig. 2). We discussed with the patient that his treatment options included, unroofing, translocation and reimplantation of the RCA, or coronary artery bypass surgery. Though translocation and reimplantation of the RCA would restore normal anatomic and physical conditions, given the presence of a proximal atherosclerotic lesion in the RCA, we decided to perform coronary artery bypass grafting of his proximal RCA with the right internal mammary artery as the choice of conduit. The proximal RCA would likely have to be ligated to eliminate competitive flow, but the final decision would be made intra-operatively after assessing occluded vs. un-occluded graft flow measurements.

**Fig. 3:**
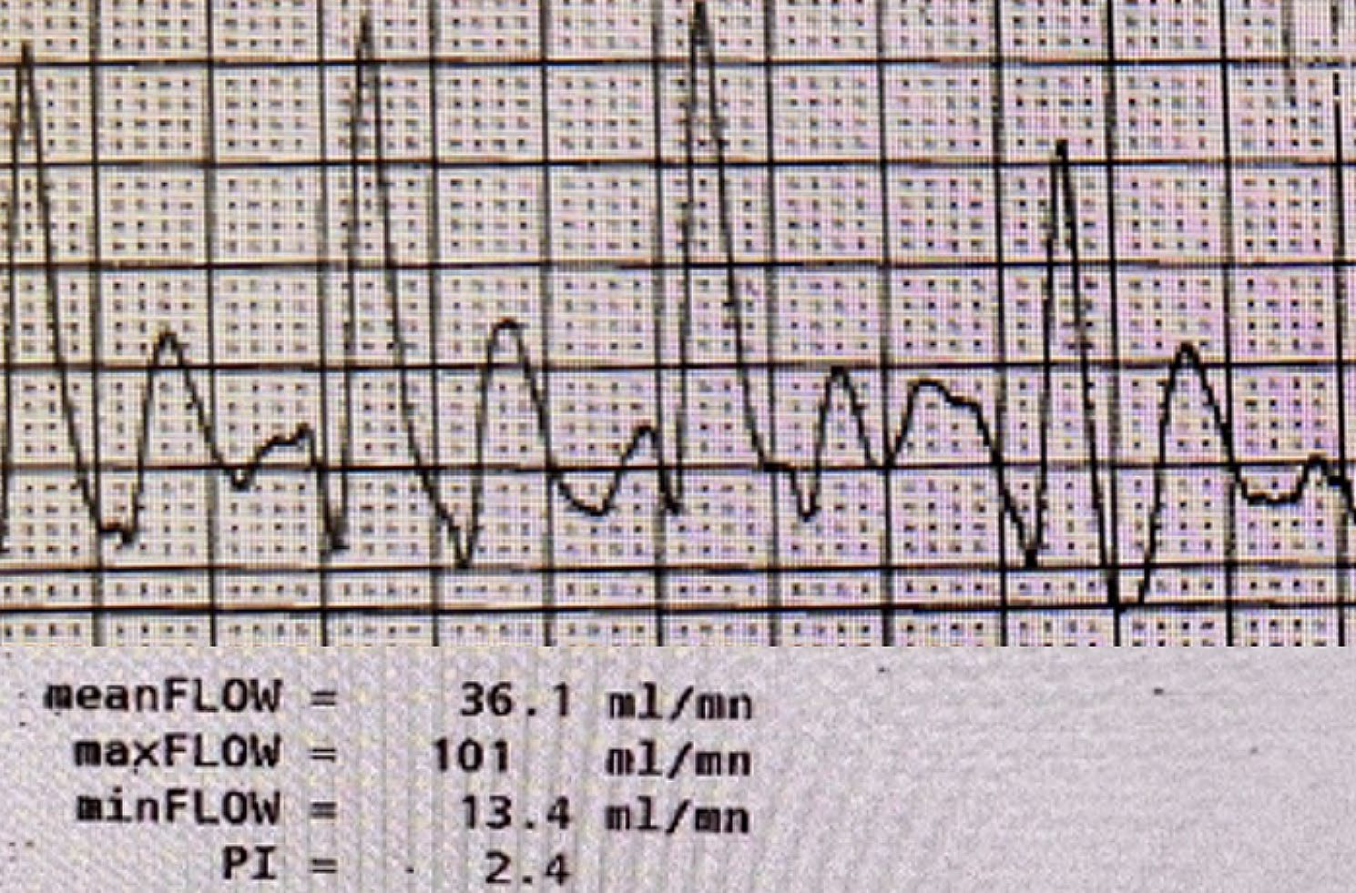
Flow probe waveform via the right internal mammary artery (RIMA) conduit obtained while the proximal RCA is occluded. PI, pulsatility index, is calculated as follows: (Maximum systolic flow - Minimum diastolic flow) / Mean flow)

We performed coronary artery bypass grafting via a median sternotomy, and cannulation of the aorta and right atrium was done for cardiopulmonary bypass. The right internal mammary artery (RIMA) was skeletonized and used as a conduit. Hypothermic cardiac arrest using del Nido Cardioplegia was performed to achieve a myocardial temperature of 10℃. The distal RCA was identified as the target (segment 2 of the RCA), and a silastic band was placed around the proximal RCA. A 2.0 mm coronary flow probe (Transonic, Ithaca, NY, USA) was used to measure flow via the RIMA with the proximal RCA occluded (101 mL/min, pulsatility index of 2.4) and un-occluded (60 mL/min, pulsatility index of 5.3). Peak flow via the graft was greater with the proximal RCA occluded, effectively eliminating any competitive flow (Fig. 3). We then ligated the proximal RCA with a 2 − 0 silk suture and three metal clips. Intra-operatively, there were no signs of ischemia or arrhythmia. The patient was successfully taken off cardiopulmonary bypass with an aortic cross-clamp time of 53 minutes. Post-operatively, he did well and was discharged on postoperative day 4 with low-dose aspirin. He continues to be asymptomatic since his procedure and has not required any hospitalizations.

## Discussion

Anomalous origin of the Coronary artery from the opposite sinus of Valsalva (ACAOS) is associated with a wide range of presentations (malignant vs. benign) and with a 1% prevalence within the general population. The most common malignant presentation is an inter-arterial course between the ascending aorta and pulmonary artery, which places the vessel at high risk of mechanical compression during strenuous activity [4]. This compression, primarily associated with a left ACAOS, can occur with a right ACAOS, resulting in sudden cardiac death, particularly in young athletes. High-risk features such as an inter-arterial course, ostial tightness, and an acute take-off angle are associated with sudden cardiac death in young athletes. ACAOS can clinically present with exercise-induced chest pain, dyspnea, or pre-syncopal episodes. Complications can result in arrhythmias and cardiac arrest [5]. Management strategies consist of medical and surgical options, with surgery recommended for patients with an anomalous artery from the left sinus or a symptomatic anomalous artery from the right sinus [6, 7]. Surgical management ranges from unroofing, right coronary translocation, and coronary artery bypass surgery with no one surgical technique shown to be superior [3, 8]. 

Unroofing involves creating an aortotomy to free the intramural segment of the RCA and creating a neo-ostium. It is the most commonly performed procedure for an anomalous anatomical origin of the RCA and a relatively low-risk procedure performed in younger populations. It is reserved for long-segment intramural anomalous RCA and does not effectively address other high-risk features such as acute angulation at take-off and an inter-arterial course. Furthermore, surgical unroofing can cause aortic regurgitation particularly when the anomalous vessel traverses below or adjacent to the commissure [9]. Translocation and reimplantation are less commonly performed and technically most challenging, as it involves repositioning the RCA orifice to its correct anatomical location. One of the main advantages of this technique is minimal manipulation of the aortic wall, valve, and root while using similar aorto-coronary anastomotic techniques such as a CABG. The presence of atherosclerosis or hypoplasia of the native RCA can limit coronary flow and make this strategy less advantageous. Lastly, CABG with either an arterial or venous conduit is reserved for older patients with acquired CAD. Since CABG is the most widely performed cardiac procedure, most surgeons are familiar with the technique of bypassing the proximal RCA. In addition, it avoids an aortotomy and manipulation of the intercoronary fissure [3].

Our patient had been intermittently symptomatic for about ten years, with further investigative testing showing an anomalous origin of the RCA from the left coronary sinus with an acute angulation of the origin and an inter-arterial course. A surgical bypass instead of a right coronary translocation or unroofing was chosen, given the patient’s age, concomitant CAD with a 70% stenotic atherosclerotic lesion of his proximal RCA, and inter-arterial course of the RCA. Though this is a flow-limiting lesion, we decided to ligate the proximal RCA as there was a significant improvement in coronary flow after test occlusion – indicating competitive flow, which can lead to conduit thrombosis. He successfully underwent a CABG with a bypass to his mid-RCA using his RIMA as a conduit. Intra-operative flow measurements via the conduit were assessed and appeared to be improved with proximal compression of the RCA. The phenomenon of competitive flow is more common in arterial conduits due to the lower conductance compared to vein graft conduits [10]. As such, we decided to suture ligate the proximal RCA with a 2 − 0 silk suture and reinforce the ligation with three large metal clips to eliminate any competitive flow.

## Conclusion

Symptomatic anomalous origin of the right coronary artery with an inter-arterial course can be treated successfully with coronary artery bypass surgery with the internal mammary artery as a conduit. Ligation of the proximal right coronary artery is essential to minimize competitive flow through the bypass graft.

## Data Availability

The data used is available upon request from the corresponding author.

## Abbreviations

ACAOS: Anomalous Coronary Arteries originating from the opposite sinus of Valsalva
CABG: Coronary Artery Bypass Grafting
RCA: Right Coronary Artery
LCA: Left Coronary Artery
